# Adaptive Sampling for Learning Gaussian Processes Using Mobile Sensor Networks

**DOI:** 10.3390/s110303051

**Published:** 2011-03-09

**Authors:** Yunfei Xu, Jongeun Choi

**Affiliations:** 1 Department of Mechanical Engineering, Michigan State University, East Lansing, MI 48823, USA; E-Mail: xuyunfei@egr.msu.edu; 2 Department of Electrical and Computer Engineering, Michigan State University, East Lansing, MI 48823, USA

**Keywords:** mobile sensor networks, Gaussian processes, adaptive sampling

## Abstract

This paper presents a novel class of self-organizing sensing agents that adaptively learn an anisotropic, spatio-temporal Gaussian process using noisy measurements and move in order to improve the quality of the estimated covariance function. This approach is based on a class of anisotropic covariance functions of Gaussian processes introduced to model a broad range of spatio-temporal physical phenomena. The covariance function is assumed to be unknown a priori. Hence, it is estimated by the maximum a posteriori probability (MAP) estimator. The prediction of the field of interest is then obtained based on the MAP estimate of the covariance function. An optimal sampling strategy is proposed to minimize the information-theoretic cost function of the Fisher Information Matrix. Simulation results demonstrate the effectiveness and the adaptability of the proposed scheme.

## 1. Introduction

In recent years, due to global climate changes, more environmental scientists are interested in the change of ecosystems over vast regions in lands, oceans, and lakes. For instance, for certain environmental conditions, rapidly reproducing harmful algal blooms in the Great Lakes can produce cyanotoxins. Besides such natural disasters, there exist growing ubiquitous possibilities of the release of toxic chemicals and contaminants in the air, lakes, and public water systems. This resulted in the rising demands to utilize autonomous robotic systems that can perform a series of tasks such as estimation, prediction, monitoring, tracking and removal of a scalar field of interest undergoing often complex transport phenomena (Common examples are diffusion, convection, and advection).

Significant enhancements have been made in the areas of mobile sensor networks and mobile sensing vehicles such as unmanned ground vehicles, autonomous underwater vehicles, and unmanned aerial vehicles. Emerging technologies have been reported on the coordination of mobile sensing agents [1–6]. Mobile sensing agents form an ad-hoc wireless communication network in which each agent usually operates under a short communication range, with limited memory and computational power. Mobile sensing agents are often spatially distributed in an uncertain surveillance environment.

The mobility of mobile agents can be designed in order to perform the optimal sampling of the field of interest. Recently in [5], Leonard *et al.* developed mobile sensor networks that optimize ocean sampling performance defined in terms of uncertainty in a model estimate of a sampled field. In [6], distributed learning and cooperative control were developed for multi-agent systems to discover peaks of the unknown field based on the recursive estimation of an unknown field. In general, we design the mobility of sensing agents to find the most informative locations to make observations for a spatio-temporal phenomenon. To find these locations that predict the phenomena best, one needs a model of the spatio-temporal phenomenon itself. In our approach, we focus on Gaussian processes to model fields undergoing transport phenomena. A Gaussian process (or kriging in geostatistics) has been widely used as a nonlinear regression technique to estimate and predict geostatistical data [7–11]. A Gaussian process is a natural generalization of the Gaussian probability distribution. It generalizes the Gaussian distribution with a finite number of random variables to a Gaussian process with an infinite number of random variables in the surveillance region. Gaussian process modeling enables us to predict physical values, such as temperature and plume concentration, at any of spatial points with a predicted uncertainty level efficiently. For instance, near-optimal static sensor placements with a mutual information criterion in Gaussian processes were proposed by [12,13]. Distributed kriged Kalman filter for spatial estimation based on mobile sensor networks are developed by [14]. A distributed adaptive sampling approach was proposed by [15] for sensor networks to find locations that maximize the information contents by assuming that the covariance function is known up to a scaling parameter. Multi-agent systems that are versatile for various tasks by exploiting predictive posterior statistics of Gaussian processes were developed by [16,17].

The motivation of our work is as follows. Even though there have been efforts to utilize Gaussian processes to model and predict the spatio-temporal field of interest, most of recent papers assume that Gaussian processes are isotropic, implying that the covariance function only depends on the distance between locations. Many studies also assume that the corresponding covariance functions are known a priori for simplicity. However, this is not the case in general as pointed out in literature [12,13,18], in which they treat the non-stationary process by fusing a collection of isotropic spatial Gaussian processes associated with a set of local regions. Hence our motivation is to develop theoretically-sound algorithms for mobile sensor networks to learn the anisotropic covariance function of a spatio-temporal Gaussian process. Mobile sensing agents can then predict the Gaussian process based on the estimated covariance function in a nonparametric manner.

The contribution of this paper is to develop covariance function learning algorithms for the sensing agents to perform nonparametric prediction based on a properly adapted Gaussian process for a given spatio-temporal phenomenon. By introducing a generalized covariance function, we expand the class of Gaussian processes to include the anisotropic spatio-temporal phenomena. The maximum a posteriori probability (MAP) estimator is used to find hyperparameters for the associated covariance function. The proposed optimal navigation strategy for autonomous vehicles minimizes the information-theoretic cost function such as D-or A-optimality criterion using the Fisher Information Matrix (or Cramér-Rao lower bound (CRLB)[19], improving the quality of the estimated covariance function. A Gaussian process with a time-varying covariance function has been proposed to demonstrate the adaptability of the proposed scheme.

This paper is organized as follows. In Section 2, we briefly review the mobile sensing network model and the notation related to a graph. A nonparametric approach to predict a field of interest based on measurements is presented in Section 3. Section 4 introduces a covariance function learning algorithm for an anisotropic, spatio-temporal Gaussian process. An optimal navigation strategy is described in Section 5. In Section 6, simulation results illustrate the usefulness of our approach and its adaptability for unknown and/or time-varying covariance functions.

The standard notation will be used in the paper. Let ℝ, ℝ_≥0_, ℤ denote, respectively, the set of real, non-negative real, and integer numbers. The positive semi-definiteness of a matrix *A* is denoted by *A* ≽ 0. Let |*B*| denotes the determinant of a matrix *B*. 𝔼 denote the expectation operator.

## 2. Mobile Sensor Networks

First, we explain the mobile sensing network and the measurement model used in this paper. Let *N_s_* be the number of sensing agents distributed over the surveillance region 𝒬 ∈ ℝ^2^. Assume that 𝒬 is a compact set. The identity of each agent is indexed by ℐ := {1, 2,⋯, *N_s_*}. Let *q_i_*(*t*) ∈ 𝒬 be the location of the *i*-th sensing agent at time *t* ∈ ℝ_≥0_. We assume that the measurement *y*(*q_i_*(*t*), *t*) of agent *i* is the sum of the scalar value of the Gaussian process *z*(*q_i_*(*t*), *t*) and sensor noise *w_i_*(*t*), at its position *q_i_*(*t*) and some measurement time *t*,

$$y\left(q_{i}\left(t\right),\;t\right):=z\left(q_{i}\left(t\right),\;t\right)+w_{i}\left(t\right).$$

The communication network of mobile agents can be represented by a graph with edges. Let *G*(*t*) = (ℐ, ℰ(*t*)) be an undirected communication graph such that an edge (*i*, *j*) ∈ ℰ(*t*) if and only if agent *i* can communicate with agent *j* ≠ *i*. We define the neighborhood of agent *i* at time *t* by *N_i_*(*t*) := {*j* | (*i*, *j*) ∈ *ℰ*(*t*), *i* ∈ ℐ}. We also define the closed neighborhood of agent *i* at time *t* by the union of its index and its neighbors, *i.e.*, *N̄_i_* (*t*) := {*i*} ∪ *N_i_*(*t*).

## 3. The Nonparametric Approach

With the spatially distributed sampling capability, agents need to estimate and predict the field of interest by fusing the collective samples from different space and time indices. We show a nonparametric approach to predict a field of interest based on measurements. We assume that a field undergoing a physical transport phenomenon can be modeled by a spatio-temporal Gaussian process, which can be used for nonparametric prediction.

Consider a spatio-temporal Gaussian process:

(1) z(s,t)∼𝒢𝒫 (μ(s,t),𝒦(s,t,s′,t′))

where *s*, *s*′ ∈ 𝒬, *t*, *t*′ ∈ ℝ_≥0_ and *μ*(*s*, *t*) denotes the mean value at location *s* and time *t*. We then propose the following generalized covariance function 𝒦(*s*, *t*, *s*′, *t*′;Ψ) with a hyperparameter vector $\Psi :={\left[\begin{array}{llll} \sigma_{f} & \sigma_{x} & \sigma_{y} & \sigma_{t} \end{array}\right]}^{T}$:

(2) $$𝒦\left(s,\;t,\;s\prime ,\;t;\Psi \right)=\sigma_{f}^{2}\;\text{exp}\;\left(-\sum_{l\in \left\{x,y\right\}}\frac{{\left(s_{l}-s_{l}^{\prime }\right)}^{2}}{{2\sigma }_{l}^{2}}\right)\;\text{exp}\;\left(-\frac{{\left(t-t\prime \right)}^{2}}{{2\sigma }_{t}^{2}}\right)$$

where *s_l_* is the *l*-th entry of *s*. {*σ_x_*, *σ_y_*} and *σ_t_* are kernel bandwidths for space and time, respectively. Equation (2) shows that points close in the measurement space and time indices are strongly correlated and produce similar values. In reality, the larger temporal distance two measurements are taken with, the less correlated they become, which strongly supports our generalized covariance function in Equation (2). This may also justify the truncation (or windowing) of the observed time series data to limit the size of the covariance matrix for reducing the computational cost.

In the case that the global coordinates are different from the local model coordinates, a similarity transformation can be used to address this issue. For instance, a rotational relationship between the model basis {*e⃗_x_*, *e⃗_y_*} and the global basis {*E⃗_x_*, *E⃗_y_*} is:

$$\left[\begin{array}{c} {\vec{e}}_{x}\\ {\vec{e}}_{y} \end{array}\right]=\left[\begin{array}{cc} \text{cos}\;\theta & \text{sin}\;\theta \\ -\text{sin}\;\theta & \text{cos}\;\theta \end{array}\right]\left[\begin{array}{c} {\vec{E}}_{x}\\ {\vec{E}}_{y} \end{array}\right]$$

where *θ* represents the angle of rotation. We can then use the following relationship to change the coordinates:

$$\{\begin{array}{c} x=X\;\text{cos}\;\theta +Y\;\text{sin}\;\theta \\ y=-X\;\text{sin}\;\theta +Y\;\text{cos}\;\theta \end{array}$$

where *x* and *y* indicate coordinates in the local basis and *X* and *Y* indicate their counterparts in the global basis. Equation (2) can then be rewritten in terms of global coordinates as

$$\begin{array}{ll} 𝒦\left(s,\;t,\;s\prime ,\;t\prime ,\;\Psi \right) & =\sigma_{f}^{2}\;\text{exp}\;\left(-\frac{{\left[\left(s_{X}-s_{X}^{\prime }\right)\;\text{cos}\;\theta +\left(s_{Y}-s_{Y}^{\prime }\right)\;\text{sin}\;\theta \right]}^{2}}{{2\sigma }_{x}^{2}}\right)\\ & \cdot \;\text{exp}\;\left(-\frac{{\left[-\left(s_{X}-s_{X}^{\prime }\right)\;\text{sin}\;\theta +\left(s_{Y}-s_{Y}^{\prime }\right)\;\text{cos}\;\theta \right]}^{2}}{{2\sigma }_{y}^{2}}\right)\;\text{exp}\;\left(-\frac{{\left(t-t\prime \right)}^{2}}{{2\sigma }_{t}^{2}}\right) \end{array}$$

where *s_X_* and *s_Y_* are the coordinates in the global basis. In this case, the parameter vector Ψ is redefined as $\Psi :={\left[\begin{array}{lllll} \sigma_{f} & \sigma_{x} & \sigma_{y} & \sigma_{t} & \theta \end{array}\right]}^{T}$.

Up to time *t_k_*, agent *i* has noisy collective data {*y*(*q_j_*(*t_m_*), *t_m_*) | *m* ∈ ℤ, *j* ∈ *N̄_i_*(*t_m_*), 1 ≤ *m* ≤ *k*}, where *N̄_i_*(*t_m_*) denotes the closed neighborhood of agent *i* at time *t_m_*. The measurements *y*(*q_j_*(*t_m_*), *t_m_*) = *z*(*q_j_*(*t_m_*), *t_m_*) + *w_j_*(*t_m_*) are taken at different positions *q_j_*(*t_m_*) ∈ 𝒬 and different times *t_m_* ∈ ℝ_≥0_. The measurements are corrupted by the sensor and communication noises represented by Gaussian white noise $w_{j}∼𝒩(0,\sigma_{w}^{2})$. For the case in which the noise level *σ_w_* is not known and needs to be estimated, the hyperparameter vector can be expanded to include *σ_w_*, *i.e.*, $\Psi :={\left[\begin{array}{lllll} \sigma_{f} & \sigma_{x} & \sigma_{y} & \sigma_{t} & \sigma_{w} \end{array}\right]}^{T}$. The column-vectorized measurements collected by agent *i* is denoted by

$$Y_{\leq k}:=\text{col}\;\left(y\left(q_{j}\left(t_{m}\right),\;t_{m}\right)|m\in 𝓁,\;j\in {\bar{N}}_{i}\left(t_{m}\right),\;1\leq m\leq k\right)$$

with a joint distribution

$$p\left(Y_{\leq k}|\Psi \right):=\frac{1}{{\left(2\pi \right)}^{n/2}|\sum_{Y_{\leq k}}{|}^{1/2}}\;\text{exp}\;\left(-\frac{1}{2}{\left(Y_{\leq k}-\mu_{Y_{\leq k}}\right)}^{T}\sum_{Y_{\leq k}}^{-1}\left(Y_{\leq k}-\mu_{Y_{\leq k}}\right)\right)$$

where *n* is the total number of observations up to time *t_k_*, *μ*_*Y*_≤*k*__ := 𝔼(*Y*_≤*k*_) is the mean vector of *Y*_≤*k*_, Σ_*Y*_≤*k*__:= 𝔼((*Y*_≤*k*_ − *μ*_*Y_≤k_*_) (*Y*_≤*k*_ − *μ*_*Y_≤*k*_*_)*^T^*) is the covariance matrix of *Y*_≤*k*_ obtained by ${\left[\sum_{Y_{\leq k}}\right]}_{\mathrm{ij}}=𝒦(s_{i},\;t_{i},\;s_{j},\;t_{j})+\sigma_{w}^{2}\delta_{\mathrm{ij}}$ in which *δ_ij_* denotes the Kronecker delta function.

If the covariance function is known a priori, the prediction of the random field *z*(*s*, *t*) at location *s* and time *t* is then obtained by

(3) $$z\left(s,\;t|t_{k}\right)\;:=z\left(s,\;t\right)|Y_{\leq k}∼𝒩\left(\hat{z}\left(s,\;t|t_{k}\right),\sigma^{2}\left(s,\;t|t_{k}\right)\right)$$

where *ẑ*(*s*, *t*|*t_k_*) := 𝔼 (*z*(*s*, *t*|*t_k_*)) is

$$\hat{z}\left(s,\;t|t_{k}\right)\;:=\mu \left(s,\;t\right)+\sum_{zY_{\leq k}}\sum_{Y_{\leq k}}^{-1}\left(Y_{\leq k}-\mu_{Y_{\leq k}}\right)$$

and the prediction error variance is

$$\sigma^{2}\left(s,\;t|t_{k}\right)\;:=\sum_{z}-\sum_{zY_{\leq k}}\sum_{Y_{\leq k}}^{-1}\sum_{Y_{\leq k}z}$$

where Σ*_z_* is the covariance of *z*, obtained by 𝒦(*s*, *t*, *s*, *t*;Ψ), $\sum_{zY_{\leq k}}=\sum_{Y_{\leq k}z}^{T}$ is the covariance matrix between *z* and *Y_≤k_*, obtained by [Σ_*zY*_≤*k*__]*_j_* = 𝒦(*s*, *t*, *s_j_*, *t_j_*; Ψ). Each agent can then predict the field of interest at any location and time with the associated uncertainty in a nonparametric way. In the next section, we present a learning approach for unknown covariance functions.

## 4. The MAP Estimate of the Hyperparameter Vector

Without loss of generality, we use a zero mean Gaussian process *z*(*s*, *t*) ∼ 𝒢𝒫(0, 𝒦(*s*, *t*, *s*′, *t*′)) for modeling the field undergoing a physical transport phenomenon. This is not a strong limitation since the mean of the posterior process is not confined to zero [11].

If the covariance function of a Gaussian process is not known a priori, mobile agents need to estimate parameters of the covariance function (Ψ) based on the observed samples. Using Bayes’ rule, the posterior *p*(Ψ*|Y*_≤*k*_) is proportional to the likelihood *p*(*Y*_≤*k*_|Ψ) times the prior *p*(Ψ), *i.e.*,

$$p\left(\Psi |Y_{\leq k}\right)\propto p\left(Y_{\leq k}|\Psi \right)p\left(\Psi \right)$$

At time *t_k_*, the maximum a posteriori (MAP) estimate Ψ̂*_k_* of the hyperparameter vector can be obtained by

(4) $$\begin{array}{ll} {\hat{\Psi }}_{k} & =\text{arg}\;\underset{\Psi }{\text{max}}\;p\left(\Psi |Y_{\leq k}\right)\\ & =\text{arg}\;\underset{\Psi }{\text{max}}\;p\left(Y_{\leq k}|\Psi \right)p\left(\Psi \right) \end{array}$$

This is equivalent to maximize the logarithm of the posterior *p*(Ψ*|Y*_≤*k*_), *i.e.*,

$${\hat{\Psi }}_{k}=\text{arg}\;\underset{\Psi }{\text{max}}\;\left(\text{ln}\;p\left(Y_{\leq k}|\Psi \right)+\text{ln}\;p\left(\Psi \right)\right)$$

The log likelihood function is given by

$$\text{ln}\;p\left(Y_{\leq k}|\Psi \right)=-\frac{1}{2}Y_{\leq k}^{T}\sum_{Y_{\leq k}}^{-1}Y_{\leq k}-\frac{1}{2}\text{ln}\;\left|\sum_{Y_{\leq k}}\right|-\frac{n}{2}\text{ln}\;2\pi$$

where *n* is the size of *Y*_≤*k*_. Notice that if no prior information is given, the MAP estimate in Equation (4) is equal to the maximum likelihood (ML) estimate.

A gradient ascent algorithm is used to find a MAP estimate of Ψ:

$${\hat{\Psi }}_{k}^{\left(i+1\right)}={\hat{\Psi }}_{k}^{\left(i\right)}+\varepsilon_{k}^{\left(i\right)}\nabla_{{\hat{\Psi }}_{k}^{\left(i\right)}}\;\text{ln}\;p\left({\hat{\Psi }}_{k}^{\left(i\right)}|Y_{\leq k}\right),\;\;\;\;\;\;\;\;i\geq 0$$

where $\varepsilon_{k}^{\left(i\right)}$ is a small positive number which can be obtained by using a backtracking line search and ∇*_x_f*(*x*) is the partial derivative of *f*(*x*) with respect to *x*. The partial derivative of the log likelihood function with respect to a hyperparameter *ψ_j_* is given by

(5) $$\begin{array}{c} \frac{\partial \;\text{ln}\;p\left(Y_{\leq k}|\Psi \right)}{{\partial \psi }_{j}}=\frac{1}{2}Y_{\leq k}^{T}\sum_{Y_{\leq k}}^{-1}\frac{{\partial \sum }_{Y_{\leq k}}}{{\partial \psi }_{j}}\sum_{Y_{\leq k}}^{-1}Y_{\leq k}-\frac{1}{2}\text{tr}\;\left(\sum_{Y_{\leq k}}^{-1}\frac{\partial \sum_{Y_{\leq k}}}{\partial \psi_{j}}\right)\\ =\frac{1}{2}\text{tr}\;\left(\left(\beta \beta^{T}-\sum_{Y_{\leq k}}^{-1}\right)\frac{{\partial \sum }_{Y_{\leq k}}}{{\partial \psi }_{j}}\right) \end{array}$$

where $\beta =\sum_{Y_{\leq k}}^{-1}Y_{\leq k}$. Alternatively, a simplex search method [20] can be used to find a MAP estimate of Ψ. This is a direct search method that does not use numerical or analytic gradients.

After finding a MAP estimate of Ψ, agents can proceed the prediction of the field of interest using Equation (3).

## 5. An Adaptive Sampling Strategy

Agents should find new sampling positions to improve the quality of the estimated covariance function in the next iteration at time *t*_*k*+1_. For instance, to precisely estimate the anisotropic phenomenon, *i.e.*, processes with different correlations along *x*-axis and *y*-axis directions, sensing agents need to explore and sample measurements along different directions.

To this end, we consider a centralized scheme. Suppose that a leader agent (or a central station) knows the communication graph at the next iteration time *t*_*k*+1_ and also has access to all measurements collected by agents. Let *Y*_*k*+1_ and *Y_≤k_* be the measurements at time *t*_*k*+1_ and the collective measurements up to time *t_k_*, respectively, *i.e.*,

$$\begin{array}{l} Y_{k+1}:=\text{col}\;\left(y\left(q_{i}\left(t_{k+1}\right),\;t_{k+1}\right)|i\in 𝒤\right),\\ Y_{\leq k}:=\text{col}\;\left(y\left(q_{i}\left(t_{m}\right),\;t_{m}\right)|m\in 𝓁,\;i\in 𝒤,\;1\leq m\leq k\right) \end{array}$$

To derive the optimal navigation strategy, we compute the log likelihood function of observations of *Y*_≤*k*+1_:

(6) $$\begin{array}{ll} 𝒧\left(Y_{\leq k+1},\Psi \right) & :=\text{ln}\;p\left(Y_{\leq k+1}|\Psi \right)\\ & =-\frac{1}{2}Y_{\leq k+1}^{T}\sum_{Y_{\leq k+1}}^{-1}Y_{\leq k+1}-\frac{1}{2}\text{ln}\left|\sum_{Y_{\leq k+1}}\right|-\frac{n_{\leq k+1}}{2}\;\text{ln}\;2\pi \end{array}$$

where *n*_≤*k*+1_ is the size of *Y*_≤*k*+1_.

Since the locations of observations in *Y*_≤*k*_ were already fixed, we represent the log likelihood function in terms of a vector of future sampling points *q̃* at time *t*_*k*+1_ only and the hyperparameter vector Ψ:

$$𝒧\left(\tilde{q},\;\Psi \right):=\text{ln}\;p\left(Y_{\leq k+1}\left(\tilde{q}\right)|\Psi \right)$$

Now consider the Fisher Information Matrix (FIM) that measures the information produced by measurements *Y*_≤*k*+1_ for estimating the hyperparameter vector at time *t*_*k*+1_. The Cramér-Rao lower bound (CRLB) theorem states that the inverse of the FIM is a lower bound of the estimation error covariance matrix [19,21]:

$$𝔼\;\left(\left({\hat{\Psi }}_{k+1}-\Psi \right){\left({\hat{\Psi }}_{k+1}-\Psi \right)}^{T}\right)\;≽\;{\text{FIM}}^{-1}$$

where Ψ̂_*k*+1_ represents the estimation of Ψ at time *t*_*k*+1_. The FIM [19] is given by

(7) $${\left[\text{FIM}\left(\tilde{q},\;\Psi \right)\right]}_{\mathrm{ij}}=-𝔼\left(\frac{\partial^{2}𝒧\left(\tilde{q},\;\Psi \right)}{{\partial \psi }_{i}{\partial \psi }_{j}}\right)$$

where the expectation is taken with respect to *p*(*Y*_≤*k*+1_ | Ψ). The analytical closed-form of FIM is given by

$${\left[\text{FIM}\left(\tilde{q},\;\Psi \right)\right]}_{\mathrm{ij}}=\frac{1}{2}\text{tr}\;\left(\sum_{Y_{\leq k+1}}^{-1}\frac{{\partial \sum }_{Y_{\leq k+1}}}{{\partial \psi }_{i}}\sum_{Y_{\leq k+1}}^{-1}\frac{{\partial \sum }_{Y_{\leq k+1}}}{{\partial \psi }_{j}}\right)$$

Since the true value of Ψ is not available, we will evaluate the FIM in Equation (7) at the currently available best estimate Ψ̂*_k_*. This has been an effective practical solution when we evaluate the FIM and estimate Ψ simultaneously [22,23]. The term due to the MAP estimation error in evaluating the FIM in Equation (7) will decrease as the number of samples increases.

We can expect that minimizing the CRLB results in a decrease of uncertainty in estimating Ψ [22]. Using the D-optimality criterion [24,25], the objective function *J* is given by

(8) $$J\left(\tilde{q},\;{\hat{\Psi }}_{k}\right):=\text{det}\;\left({\text{FIM}}^{-1}\left(\tilde{q},\;{\hat{\Psi }}_{k}\right)\right)$$

Minimizing *J* in Equation (8) corresponds to minimizing the volume of the ellipsoid which represents the maximum confidence region for the estimated hyperparameters. However, if one hyperparameter has a much larger variance compared to the others, minimizing the volume may not be very useful [25]. As an alternative, the A-optimality which minimizes the sum of the variances may be used. The objective function *J* based on the A-optimality criterion is

(9) $$J\left(\tilde{q},\;{\hat{\Psi }}_{k}\right):=\text{tr}\;\left({\text{FIM}}^{-1}\left(\tilde{q},\;{\hat{\Psi }}_{k}\right)\right)$$

A control law for the mobile sensor network can be formulated as follows:

(10) $$q\left(t_{k+1}\right)=\text{arg}\underset{\tilde{q}\in 𝒬}{\text{min}}\;J\;\left(\tilde{q},\;{\hat{\Psi }}_{k}\right)$$

A gradient descent strategy can be used to find the next optimal sampling positions:

(11) $${\tilde{q}}^{\left(i+1\right)}={\tilde{q}}^{\left(i\right)}-\alpha^{\left(i\right)}\nabla_{{\tilde{q}}^{\left(i\right)}}\;J\;\left({\tilde{q}}^{\left(i\right)},\;{\hat{\Psi }}_{k}\right)$$

where *α*^(*i*)^ is a small positive number which can be obtained by using a backtracking line search. Alternatively, a control law for the mobile sensor network can be formulated as follows:

$$q\left(t_{k+1}\right)=\text{arg}\underset{\tilde{q}\in \Pi_{i=1}^{N_{s}}Q_{i}}{\text{min}}J\left(\tilde{q},{\hat{\Psi }}_{k}\right)$$

where

$$Q_{i}=q_{i}\left(t_{k}\right)+\prod_{j=1}^{n_{d}}\left[-\delta_{j}\;\delta_{j}\right]$$

where *n_d_* = 2 denotes the dimension of the surveillance region 𝒬 and *δ_j_* is the maximum distance for each agent to move in *x* and *y* directions.

However, optimization on ln *p*(*Y*_≤*k*+1_|Ψ) in Equation (6) and *J*(*q̃*, Ψ̂*_k_*) in Equation (8) or (9) can be numerically costly due to the increasing size of Σ_*Y*_≤*k*__. One way to deal with this problem is to use a truncated date set

$$Y_{k-\delta \leq ,\leq k}:=\text{col}\;\left(y\left(s_{j}\left(t_{m}\right),\;t_{m}\right)|m\in 𝕑,\;j\in 𝒤,\;k-\delta \leq m\leq k\right)$$

instead of using *Y*_≤*k*_. In addition, this approach based on the truncated observations can be viewed as a strategy to deal with a slowly time-varying parameter vector Ψ, which will further investigated in Section 6.2.

The overall protocol for the sensor network is summarized as in Table 1.

## 6. Simulation Results

In this section, we evaluate the proposed approach for a spatio-temporal Gaussian process (Section 6.1) and an advection-diffusion process (Section 6.3). For both cases, we compare the simulation results using the proposed optimal sampling strategy with results using a benchmark random sampling strategy. In this random sampling strategy, each agent was initially randomly deployed in the surveillance region. At each time step, the next sampling position for agent *i* is generated randomly with the same mobility constraint, *viz.* a random position within a square region with length 2 centered at the current position *q_i_*. For fair comparison, the same values were used for all other conditions. In Section 6.2, our approach based on truncated observations has been applied to a Gaussian process with a time-varying covariance function to demonstrate the adaptability of the proposed scheme.

### 6.1. A Spatio-Temporal Gaussian Process

**Figure 1.**
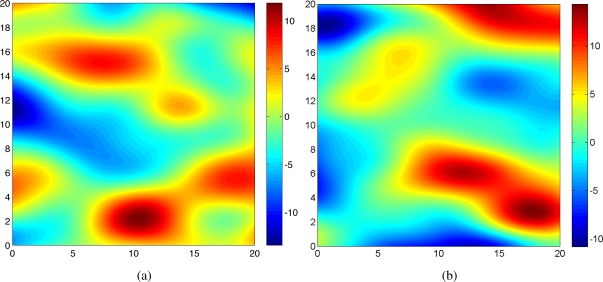
Snap shots of the realized Gaussian process at **(a)** *t* = 1 and **(b)** *t* = 20.

We apply our approach to a spatio-temporal Gaussian process. The Gaussian process was numerically generated for the simulation [11]. The hyperparameters used in the simulation were chosen such that $\Psi ={\left[\begin{array}{lllll} \sigma_{f} & \sigma_{x} & \sigma_{y} & \sigma_{t} & \sigma_{w} \end{array}\right]}^{T}={\left[\begin{array}{lllll} 5 & 4 & 2 & 8 & 0.5 \end{array}\right]}^{T}$. Two snap shots of the realized Gaussian random field at time *t* = 1 and *t* = 20 are shown in Figure 1. In this case, *N_s_* = 5 mobile sensing agents were initialized at random positions in a surveillance region $𝒬=[\begin{array}{ll} 0 & 20 \end{array}]\times [\begin{array}{ll} 0 & 20 \end{array}]$. The initial values for the algorithm were given to be $\Psi^{\left(0\right)}={\left[\begin{array}{lllll} 1 & 10 & 10 & 1 & 0.1 \end{array}\right]}^{T}$. A prior of the hyperparameter vector has been selected as

$$p\left(\Psi \right)=p\left(\sigma_{f}\right)p\left(\sigma_{x}\right)p\left(\sigma_{y}\right)p\left(\sigma_{t}\right)p\left(\sigma_{w}\right)$$

where *p*(*σ_f_*) = *p*(*σ_x_*) = *p*(*σ_y_*) = *p*(*σ_t_*) = Γ(5, 2), and *p*(*σ_w_*) = Γ(5, 0.2). Γ(*a*, *b*) is a Gamma distribution with mean *ab* and variance *ab*^2^ in which all possible values are positive. The gradient method was used to find the MAP estimate of the hyperparameter vector.

For simplicity, we assumed that the global basis is the same as the model basis. We considered a situation where at each time, measurements of agents are transmitted to a leader (or a central station) that uses our Gaussian learning algorithm and sends optimal control back to individual agents for next iteration to improve the quality of the estimated covariance function. The maximum distance for agents to move in one time step was chosen to be 1 for both *x* and *y* directions. The A-optimality criterion was used for optimal sampling.

**Figure 2.**
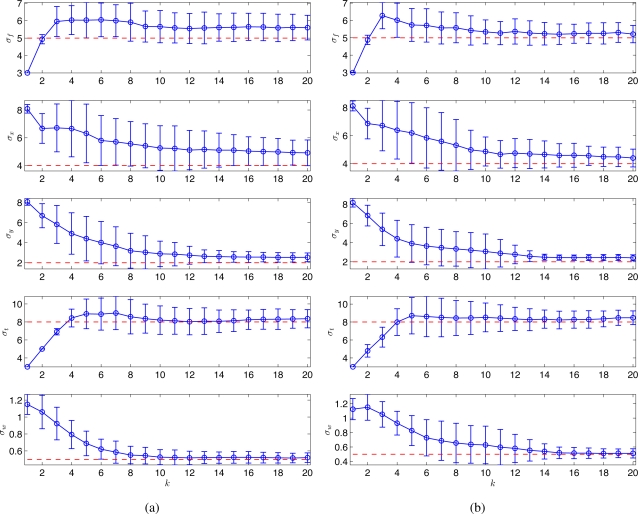
Monte Carlo simulation results (100 runs) for a spatio-temporal Gaussian process using **(a)** the random sampling strategy, and **(b)** the adaptive sampling strategy. The estimated hyperparameters are shown in blue circles with error-bars. The true hyperparameters that used for generating the process are shown in red dashed lines.

For both proposed and random strategies, Monte Carlo simulations were run for 100 times and the statistical results are shown in Figure 2. The estimates of the hyperparameters (shown in circles and error bars) tend to converge to the true values (shown in dotted lines) for both strategies. As can be seen, the proposed scheme (Figure 2(a)) outperforms the random strategy (Figure 2(b)) in terms of the A-optimality criterion.

**Figure 3.**
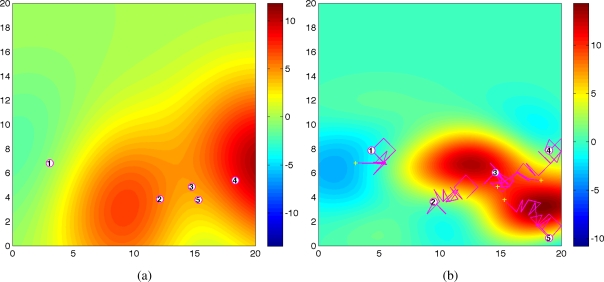
The predicted fields along with agents’ trajectories at **(a)** *t* = 1 and **(b)** *t* = 20.

Figure 3 shows the predicted field along with agents’ trajectories at time *t* = 1 and *t* = 20 for one trial. As shown in Figure 1(a) and Figure 3(a), at time *t* = 1, the predicted field is far from the true field due to the inaccurate hyperparameters estimation and small number of observations. As time increases, the predicted field will be closer to the true field due to the improved quality of the estimated the covariance function and the cumulative observations. As expected, at time *t* = 20, the quality of the predicted field is very well near the sampled positions as shown in Figure 3(b). With 100 observations, the running time is around 30*s* using Matlab, R2008a (MathWorks) in a PC (2.4 GHz Dual-Core Processor). No attempt has been made to optimize the code. After converging to a good estimate of Ψ, agents can switch to a decentralized configuration and collect samples for other goals such as peak tracking and prediction of the process [6,16,17].

### 6.2. Time-Varying Covariance Functions

To illustrate the adaptability of the proposed strategy to time-varying covariance functions, we introduce a Gaussian process defined by the following covariance function. The time-varying covariance function is modeled by a time-varying weighted sum of two known covariance functions 𝒦_1_(·) and 𝒦_2_(·) such as

(12) $$𝒦\left(\cdot \right)=\lambda \left(t\right){𝒦}_{1}\left(\cdot \right)+\left(1-\lambda \left(t\right)\right){𝒦}_{2}\left(\cdot \right)$$

where *λ*(*t*) ∈ [0, 1] is a time-varying weight factor that needs to be estimated. In the simulation study, 𝒦_1_(·) is constructed with *σ_f_* = 1, *σ_x_* = 0.2, *σ_y_* = 0.1, *σ_t_* = 8, and *σ_w_* = 0.1; and 𝒦_2_(·) is with *σ_f_* = 1, *σ_x_* = 0.1, *σ_y_* = 0.2, *σ_t_* = 8, and *σ_w_* = 0.1. This Gaussian process defined in (12) with theses particular 𝒦_1_ and 𝒦_2_ effectively models hyperparameter changes in *x* and *y* directions.

**Figure 4.**
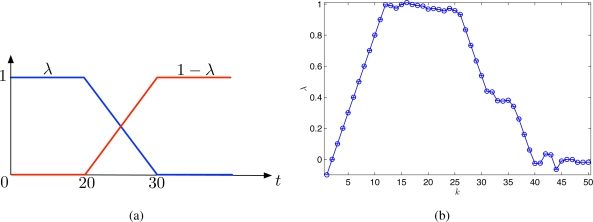
**(a)** The weighting factor *λ*(*t*) and **(b)** the estimated *λ*(*t*).

To improve the adaptability, the mobile sensor network uses only observations sampled during the last 20 iterations for estimating *λ*(*t*) online. The true *λ*(*t*) and the estimated *λ*(*t*) are shown in Figure 4(a,b), respectively. From Figure 4, it is clear that the weighting factor *λ*(*t*) can be estimated accurately after some delay about 5–8 iterations. The delay is due to using the truncated observations that contain past observations since the time-varying covariance function changes continuously in time.

### 6.3. Fitting a Gaussian Process to an Advection-Diffusion Process

**Figure 5.**
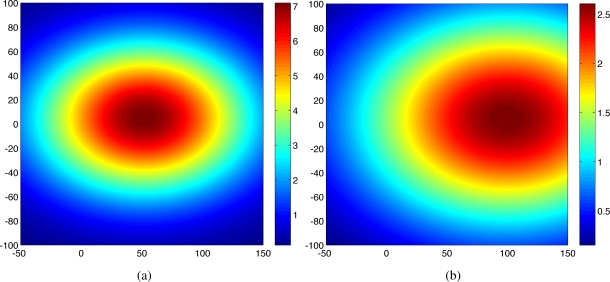
Snap shots of the advection-diffusion process at **(a)** *t* = 1 and **(b)** *t* = 20.

We apply our approach to a spatio-temporal process generated by physical phenomena (advection and diffusion). This work can be viewed as a statistical modeling of a physical process, *i.e.*, as an effort to fit a Gaussian process to a physical advection-diffusion process in practice. The advection-diffusion model developed in [26] was used to generate the experimental data numerically. An instantaneous release of *Qkg* of gas occurs at a location (*x*_0_, *y*_0_, *z*_0_). This is then spread by the wind with mean velocity $u={\left[\begin{array}{lll} u_{x} & 0 & 0 \end{array}\right]}^{T}$ Assuming that all measurements are recorded at a level *z* = 0, and the release occurs at a ground level (*i.e.*, *z*_0_ = 0), the concentration *C* at an arbitrary location (*x*, *y*, 0) and time *t* is described by the following analytical solution [27]:

(13) $$C\left(x,\;y,\;0,\;t\right)=\frac{Q\;\text{exp}\;\left(-\frac{{\left(\Delta x-u\Delta t\right)}^{2}}{{4K}_{x}\Delta t}-\frac{{\Delta y}^{2}}{{4K}_{y}\Delta t}\right)}{{4\pi }^{\frac{3}{2}}{\left(K_{x}K_{y}K_{z}\right)}^{\frac{1}{2}}{\left(\Delta t\right)}^{\frac{3}{2}}}$$

where Δ*x* = *x* − *x*_0_, Δ*y* = *y* − *y*_0_, and Δ*t* = 5(*t* − 1) + *t*_0_. The parameters used in the simulation study are shown in Table 2. Notice that this process generates an anisotropic concentration field with parameters *K_x_* = 20*m*^2^*/min* and *K_y_* = 10*m*^2^*/min* as in Table 2. The fields at time *t* = 1 and *t* = 20 are shown in Figure 5. Notice the center of the concentration moved. In this case, *N_s_* = 5 mobile sensing agents were initialized at random positions in a surveillance region $𝒬=[\begin{array}{ll} -50 & 150 \end{array}]\times [\begin{array}{ll} -100 & 100 \end{array}]$.

The initial values for the algorithm was chosen to be $\Psi^{\left(0\right)}={\left[\begin{array}{lll} 100 & 100 & 100 \end{array}\right]}^{T}$ where we assumed *σ_f_* = 1 and *σ_w_* = 0.1. For this application, we did not assume any prior knowledge about the covariance function. Hence, the MAP estimator was the same as the ML estimator. The gradient method was used to find the ML estimate.

We again assumed that the global basis is the same as the model basis and assumed all agents have the same level of measurement noises for simplicity. In our simulation study, agents start sampling at *t*_0_ = 100*min* and take measurements at time *t_k_* with a sampling time of *t_s_* = 5*min* as in Table 2.

**Figure 6.**
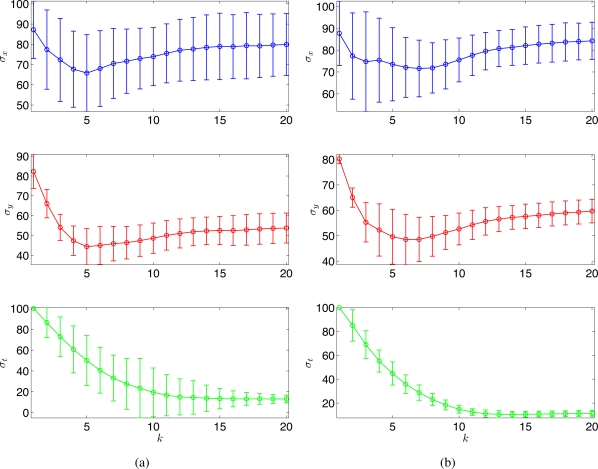
Simulation results (100 runs) for a advection-diffusion process. The estimated hyperparameters with **(a)** random sampling and **(b)** optimal sampling.

Monte Carlo simulations were run for 100 times, and Figure 6 shows the estimated *σ_x_*, *σ_y_*, and *σ_t_* with (a) the random sampling strategy and (b) the optimal sampling strategy, respectively. With 100 observations, the running time at each time step is around 20*s* using Matlab, R2008a (MathWorks) in a PC (2.4 GHz Dual-Core Processor). No attempt has been made to optimize the code. As can be seen in Figure 6, the estimates of the hyperparameters tend to converge to similar values for both strategies. Clearly, the proposed strategy outperforms the random sampling strategy in terms of the estimation error variance.

## 7. Summary

In this paper, we presented a novel class of self-organizing sensing agents that learn an anisotropic, spatio-temporal Gaussian process using noisy measurements and move in order to improve the quality of the estimated covariance function. The MAP estimator was used to estimate the hyperparameters in the unknown covariance function and the prediction of the field of interest was obtained based on the MAP estimates. An optimal navigation strategy was proposed to minimize the information-theoretic cost function of the Fisher Information Matrix for the estimated hyperparameters. The proposed scheme was applied to both a spatio-temporal Gaussian process and a true advection-diffusion field. Simulation study indicated the effectiveness of the proposed scheme and the adaptability to time-varying covariance functions. The trade-off between a precise estimation and computational efficiency using truncated observations will be studied in the future work.

## Figures and Tables

**Table 1.** An adaptive sampling strategy for mobile sensor networks.

Learning: At time *t_k_*, the sensor network updates Ψ̂*_k_* using a MAP estimate Equation (4) for a data set *Y*_≤*k*_. Start this MAP optimization with the initial point Ψ̂_*k*−1_. Prediction: For given *Y*_≤*k*_ and Ψ̂*_k_*, agents can compute prediction at any point and time using Equation (3), *i.e.*, *p*(*z*(*s*, *t*)*|Y*_≤*k*_; Ψ̂*_k_*). Sampling: Based on {Ψ̂*_k_*, *Y*_≤*k*_}, the sensor network computes the control Equation (10) in order to maximize *J*(*q̃*, Ψ̂*_k_*). Update the positions of agents accordingly and collect measurements at time *t*_*k*+1_. Repeat the steps 1–3 until Ψ converges.

**Table 2.** Parameters used in simulation.

Parameter	Notation	Unit	Value
Number of agents	*N_s_*	-	5
Sampling time	*t_s_*	min	5
Initial time	*t*_0_	min	100
Gas release mass	*Q*	kg	10^6^
Wind velocity in *x* axis	*u_x_*	m/min	0.5
Eddy diffusivity in *x* axis	*K_x_*	m^2^/*min*	20
Eddy diffusivity in *y* axis	*K_y_*	m^2^/*min*	10
Eddy diffusivity in *z* axis	*K_z_*	m^2^/*min*	0.2
Location of explosion	*x*_0_	m	2
Location of explosion	*y*_0_	m	5
Location of explosion	*z*_0_	m	0
Sensor noise level	*σ_w_*	kg/m^3^	0.1
